# Acupuncture for chronic low back pain: protocol for a multicenter, randomized, sham-controlled trial

**DOI:** 10.1186/1471-2474-11-118

**Published:** 2010-06-14

**Authors:** Jun-Hwan Lee, Hi-Joon Park, Hyangsook Lee, Im Hee Shin, Mi-Yeon Song

**Affiliations:** 1 Department of Oriental Rehabilitation Medicine, College of Oriental Medicine, Kyung Hee University, 1 Hoegi-dong, Dongdaemun-gu, Seoul 130-701, Republic of Korea; 2 Department of Meridian and Acupoints, College of Oriental Medicine, Kyung Hee University, 1 Hoegi-dong, Dongdaemun-gu, Seoul 130-701, Republic of Korea; 3 Department of Medical Statistics, School of Medicine, Catholic University of Daegu, 3056-6 Daemyung 4 Dong, Daegu, Republic of Korea

## Abstract

**Background:**

Use of acupuncture has widely increased in patients with chronic low back pain. However, the evidence supporting its efficacy remains unclear. In this article, we report the design and the protocol of a multi-center randomized sham-controlled trial to treat chronic low back pain. Our goal is to verify the effect of acupuncture on chronic low back pain.

**Methods/Design:**

This study is a multi-center randomized sham-controlled trial with 2 parallel arms. Participants included in the study met the following criteria: 1) low back pain lasting for at least the last 3 months, 2) a documented ≥ 5 points on a 10 cm visual analog scale for bothersomeness of low back pain at the time of screening and 3) between 18 and 65 years of age. Participants were blinded to the real and sham acupuncture treatments. The real acupuncture treatment group received real acupuncture 2 times a week, during a total of 12 sessions over 6 weeks. The control group received sham acupuncture during the same period. In order to assess the primary and secondary outcome measures, the participants were asked to fill out a questionnaire at the baseline and 6, 8, 12 and 24 weeks after starting the treatments. The primary outcome was measured using the visual analog scale for bothersomeness of low back pain at 8 weeks after the initiation of treatments.

**Discussion:**

The result of this trial (which will be available in 2010) will demonstrate the efficacy of using acupuncture to treat chronic low back pain.

**Trial registration:**

This study is registered with the U.S. National Institutes of Health Clinical Trials registry: NCT00815529

## Background

Chronic low back pain (LBP) is one of the most important public health problems, as well as one of the main causes of disability among adults of working age [1]. More than 50% of adults are bothered by back pain each year and over 70% of adults have suffered from back pain at some time in their lives [2,3].

Although a wide range of standard treatments are available [4], back pain patients are often dissatisfied with conventional medical care [5]. Acupuncture is one of the frequently used methods in patients with low back pain [6]. Especially in Korea, where acupuncture has a much longer tradition than in other countries, low back pain is the first reason that people consult with the Korean Medicine Doctor (KMD) for acupuncture treatment [7]. A number of randomized controlled trials (RCTs) on acupuncture for LBP have already been published [8,9]. However, conclusions regarding the efficacy and effectiveness of acupuncture for this common problem remains contradictory [8,9]. Most of these equivocal results are due to low methodological quality, small sample size, and other factors such as inherent difficulties in the use of controls [8-10]. Acupuncture is complicated to evaluate because it is difficult to isolate the characteristic or specific effects of the technique from the non-specific ones [11]. Some reviews have noted the poor quality of research in this area and have insisted on scientifically rigorous studies [8-10].

In this paper, we propose a randomized, sham-controlled study of patients with chronic low back pain, with a predetermined sample size and appropriate follow-up, which will enable us to investigate the efficacy of acupuncture on chronic low back pain.

## Methods & Design

### Overview

**Figure 1:**
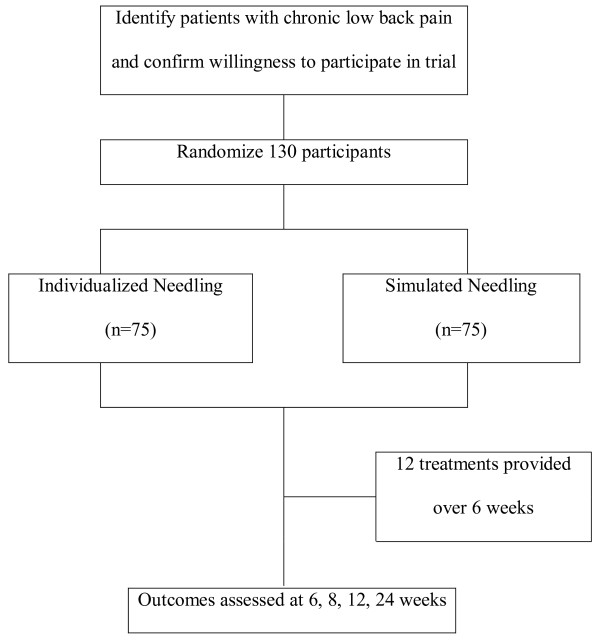
**Study sequence; Process of recruitment, randomization to treatment, and outcome assessment**.

This study is a multicenter, randomized, non-penetrating sham-controlled study. The trial was conducted in the following three hospitals after obtaining permission from the Institutional Review Boards of three institutions: East-West Neo Medical Center of Kyung Hee University, Oriental Medicine Hospital of Sang Ji University, and Kyung Won Incheon Oriental Medicine Hospital. The patients were blinded to group allocation. The outcome assessment and the statistical analysis were performed by professionals who were blinded to the assignment of patients to either real or sham acupuncture (Figure 1).

### Study duration

October 2008-June 2010

### Participants

Patients aged between 18 and 65 years with non-radicular chronic low back pain of mechanical origin were recruited.

#### Inclusion criteria

Inclusion criteria were developed mainly to exclude patients with specific or complicated low back pain.

• Between 18 and 65 years of age

• Low back pain lasting for at least the previous 3 months

• ≥ 5 points on a 10-cm visual analog scale for bothersomeness of low back pain at the time of screening

• Intact on neurological examination (e.g., lumbosacral nerve function, deep tendon reflexes, plantar response, voluntary muscle activation, and sensory function)

• Non-specific, uncomplicated low back pain, i.e., ICD-10 (International Classification of Diseases-10) codes:

M51.3   Other specified intervertebral disc degeneration

M54.5   Low back pain

M54.8   Other dorsalgia

M54.9   Dorsalgia, unspecified

S33.5   Sprain and strain of lumbar spine

S33.6   Sprain and strain of sacroiliac joint

S33.7   Sprain and strain of other and unspecified parts of lumbar spine and pelvis

• Participation agreement and signed informed consent

#### Exclusion criteria

• Radicular pain

• Pain mainly below the knee, as clinical outcome is likely to vary

• Serious spinal disorders, including malignancy, vertebral fracture, spinal infection, inflammatory spondylitis, and cauda equina compression

• Patients who previously had spinal surgery or are scheduled to have one during the study

• Other chronic diseases that could interfere with acupuncture treatment effects, including cardiovascular disease, diabetic neuropathy, fibromyalgia, rheumatoid arthritis, dementia and epilepsy

• Chief musculoskeletal pain other than back pain

• Acupuncture treatment for low back pain during the previous month

• Conditions where acupuncture might not be safe, including clotting disorders, administration of an anticoagulant agent, pregnancy, and seizure disorders

• Severe psychiatric or psychological disorder

• Inability to read and write Korean

• Pending lawsuits/receiving compensation due to low back pain

• Patient taking corticosteroids, narcotics, muscle relaxant or herbal medicine to treat low back pain or any medication considered to be inappropriate by the investigator

• Refusal to participate in the trial or informed consent

### Recruitment

Participants were recruited through advertisements in local newspapers, the hospital monthly magazine, on a website and on bulletin boards. If patients were interested in participating, they were asked to answer questions to determine eligibility. If eligible, they were guided through the informed consent process. After written consent was obtained, a study researcher administered the baseline questionnaire followed by random allocation to either the real or sham acupuncture group. After randomization, the interviewer scheduled the treatment procedure.

### Randomization procedures

The patients were randomized per center in order to give each investigator the same chance to use individual, standard, and sham acupuncture to control the selection bias of the trial. The random code for randomization was generated by the medical statistician and was kept by a clinician who did not contact patients.

Randomization was performed only after a subject was confirmed to be eligible and written informed consent was obtained. Subject details were recorded and a treatment arm and randomization number were allocated to the subject, and both of these were recorded in the subject's hospital file. The randomization form will be completed and returned to the principal investigator.

In order to ensure balance within the two groups, blocked randomization was employed. This was made possible by using computer-generated random numbers from SAS software or other computer software. Different block sizes can be used. For a block of four, the possible assignment orders are AABB, ABAB, ABBA, etc. If there is an important predictor of outcome, stratified block randomization is available. For example, gender may be a strong predictor, and then block randomization within each of the two strata, male and female can be performed. The blinding credibility of the real and sham acupuncture treatments were evaluated at the baseline and at the end of the 6-week treatment.

### Education of acupuncturists

Licensed Korean Medicine Doctors (KMDs) who specialize in Korean Rehabilitation Medicine from the three participating hospitals should take the educational course to strictly adhere to the study protocol and to be familiar with administering study treatments; all participating KMDs underwent intensive and customized training for a full understanding of the "sham acupuncture" procedure, including details such as acupuncture points, depth, and manipulation. They were also trained to administer real acupuncture using a sham needle device.

#### Treatment details

Both real and sham acupuncture groups received a total of 10-12 acupuncture sessions (approximately two times a week, for 6 weeks). In the real acupuncture group, disposable sterile needles (40 mm × 0.25 mm) were used (Dong-bang Acupuncture Inc., Seoul, Korea), while non-penetrating sham needles with identical gauge were used in the sham control group (Acuprime Co., Ltd, UK). At the time of the first acupuncture session, all participants received an "exercise manual for low back pain patients" from the spine center of East-West Neo Medical Center of Kyung Hee University and were encouraged to do exercises according to the manual during the whole treatment period. This manual includes information on physical activity, exercise and appropriate life-style modification.

##### • Real acupuncture

A pre-defined, individualized acupuncture prescription in accordance with the characteristics of pain was employed. For this study, we developed a flexible treatment protocol by selecting a group of points that were predefined in every case, allowing the participating KMD some flexibility in the choice according to the diagnosis. The predefined points were carefully selected by a process of consensus with participating KMDs who were all experienced in low back pain. When we treated the patients in this research, we chose acupuncture points after the process of the meridian pattern identification. To treat low back pain, we assumed 3 types of meridian pattern identification according to the pain location (A: Gallbladder meridian pattern, B: Bladder meridian pattern, C: Mixed pattern).

The acupuncture points included: Shenshu (BL23), Qihaishu (BL24), Dachangshu (BL25), Yimmen (BL37), Weizhong (BL40), Wangu (GB12), Daimai (GB26), Huantiao (GB30), Yanglingquan (GB34), Zulinqi (GB41), Dicang (ST4), Zusanli (ST36), Fushe (SP13), Fujie (SP14), Yaoyangguan (GV3), Mingmen (GV4), Xuanshu (GV5), Shenting (GV24) and Shuigou (GV26).

Acupuncture treatment was given using a sterile, disposable stainless steel needle (40 mm × 0.25 mm) with the aid of the tube of a sham acupuncture needle device. After the skin sterilization, the needles were inserted perpendicular to a depth of 5-20 mm with the patient lying down and blindfolded, followed by bidirectional rotation to induce *Deqi *sensation. The needles were left in place for 15-20 minutes.

As the real acupuncture group received treatment that reflects everyday practice, the acupuncture points were varied from visit to visit when the symptoms or pain changed.

##### • Sham acupuncture

Non-penetrating sham needles by Park et al. (Acuprime Co., Ltd, UK)[12] were used. Eight pre-defined points in the lower back, which are non-traditional acupuncture points, were used after skin sterilization, with patients lying down; 1 cm below from Weiyang (BL39), 1 cm lateral to Ganshu (BL18), 1 cm lateral to Pishu (BL20), and 2 cm above from Huantiao (GB30), all bilaterally. The technique did not vary in any way from that performed in the real acupuncture group, except for the usage of a semi-blunted needle. The sham needles were left in for 15-20 minutes of the session, as was done in the real acupuncture group.

### Outcome measurements

Patient completed a series of measurements to assess back pain-related dysfunction, pain, quality of life, and back pain-related costs.

At the screening visit, patients were asked to fill out a questionnaire regarding age, gender, marital status, residence, occupation, and education. Medical history was also taken before physical check-ups and it was determined whether the patient would be included or not. The baseline (T0), 4 (T1), 6 (T2), 8 (T3), 12 (T4), and 24 (T5) week follow-up measurements are summarized in Table 1.

**Table 1:** Schedule for data collection; outcome measures per visits

Measures	Baseline (0-week)	4-week	End of treatment (6-week)	8-week	12-week	24-week
Sociodemographic characteristics	X					
Back pain history	X					
X-ray	X			X		
VAS for bothersomeness of low back pain	X	X	X	X	X	X
VAS for pain intensity	X	X	X	X	X	X
Oswestry disability Index (ODI)	X		X	X	X	X
General health status (SF-36)	X		X	X	X	X
EuroQol-5D (EQ-5D)	X		X	X	X	X
Beck Depression Inventory (BDI)	X		X	X	X	X
Medication use	X		X	X		
Costs of back care	X		X	X		X
Adverse experiences*	X	X	X			
Credibility test†	X		X			
Acupuncture experience (In-depth interview)§				X		

*, At every single visit; †, After 1st treatment and at the end of treatment (6-week); §, 10 randomly chosen participants (5 from acupuncture group and 5 from sham group) for each hospital, thus 30 in total.

#### Primary outcome measures

The primary outcome was measured using the visual analogue scale (VAS) for bothersomeness of low back pain. Since our intension was not to simply evaluate the severity of pain, but also to understand the impact of chronic low back pain on the patients' life, we used the VAS not for pain intensity, but for bothersomeness of low back pain, as a primary outcome measurement. The patient was asked to mark, on a 10-cm VAS (0, absence of bothersomeness; 10, the worst bothersomeness imaginable), the degree of bothersomeness due to low back pain experienced within the most recent one week from the day the assessment was performed. This measurement has substantial validity; it is highly correlated with measures of function and other outcome measures[13]. Bothersomeness of low back pain was measured at baseline, and on the 4, 6, 8, 12, and 24-week follow-ups. The primary endpoint was the 8 week follow-up, i.e. 2 weeks after completion of the 10-12 sessions of acupuncture treatment.

#### Secondary outcome measures

There is ample evidence supporting the validity of the visual analogue scale (VAS) for pain intensity. Many studies have demonstrated the validity of its construct and its reliability [14,15]. This is a fast and straightforward method for evaluating the subjective intensity of pain. Pain intensity was measured in the same fashion as VAS for bothersomeness (0, absence of pain; 10, the worst pain imaginable) at baseline, 4, 6, 8, 12 and 24 weeks after beginning of treatment.

The Oswestry Disability Index (ODI) [16] was used to measure back pain-related dysfunction. ODI includes 10 questions about daily activities including pain intensity, personal care, lifting, walking, sitting, standing, sleeping, sexual life, social life, and traveling. Each question is rated on a 0 to 5 point scale and the lower the score, the less disabled. It takes 5 minutes to complete and the validated Korean version of ODI [17] was administered at baseline, 6, 8, 12, and 24-week follow-ups.

Health-related quality of life was measured at baseline, and at 6, 8, 12, and 24-week follow-ups, using the well-validated SF-36 [18], which has been recommended for back pain studies. The SF-36 measures 8 health domains: 1) limitations in physical activity because of health problem; 2) limitations in social activities because of physical or emotional problem; 3) limitations in usual role activities because of physical health problems; 4) bodily pain; 5) general mental health (psychological distress and well-being); 6) limitations in usual activities because of emotional problems; 7) vitality (energy and fatigue); and 8) general health perceptions. The higher score means better general health status. In our study, the validated Korean version of SF-36 [19] was analyzed.

Patients completed the EuroQol 5-Dimension (EQ-5D) [20] at baseline, and at the 6, 8, 12 and 24-week follow-ups. EQ-5D is a generic questionnaire that measures the quality of life regarding personal health. It consists of two parts: in the first part, the patient evaluates in a descriptive way his/her health state, with respect to 5 dimensions, namely mobility, personal care, daily activities, pain/discomfort and anxiety/depression. Each dimension is scored from one to three, and so the best possible health profile is 11111 and the worst is 33333. In the second part, the patient rates on a VAS from 0, the worst imaginable state of health to 100, the best imaginable state of health, his/her overall state of health on the day the questionnaire is completed. The two scores are complementary. EQ-5D has an index of reference value of possible health profiles ranging from a value of one (the best state of health) to zero (death). Thus, we sought to combine these results with the years of life in order to calculate the years of life adjusted for health-related quality of life. Using this approach, as well as analyzing cost-effectiveness, we performed a cost-utility analysis. A validated Korean version of EQ-5D [21] was administered in this study.

The Korean version of Beck's Depression Inventory (BDI) [22] is a 21-item self-administered questionnaire. It provides a quantitative measure of depressive symptoms. Each item has a 0-3 response format, giving a theoretical maximum score of 63. The following cut-off scores are recommended: scores 0-9 little, if any, depression, 10-18 mild depression, 19-29 moderate depression, and 30-63 severe depression. The psychometric quality of the questionnaire is very good [23]. The validated Korean version of the credibility test, which was first proposed by Vincent [24], was used to assess the credibility of real and sham acupuncture treatments at the end of the 6-week treatment. The patients rated the credibility of the treatment they were given by answering 4 questions on a numeric rating scale, with 0 for not at all and 6 for maximal agreement: 1) improvement expected; 2) recommendation to others; 3) treatment logical; and 4) effective also for other diseases.

### Protection of subjects and assessment of safety

Any adverse experiences at any clinic visit during the 12 sessions were monitored. The research team monitored serious adverse experiences, which were defined as treatment-related experiences.

### Stopping rules

The trial would have been stopped if the principle investigator believed there was an unacceptable risk of serious adverse events in one of more of the treatment arms.

### Sample size determination

Generally, a level of significance of α = 0.05 and a power of 1-ß = 0.80 were used. For two arms (control - sham acupuncture, experimental - real acupuncture), we considered the two sample t-test model.

Experimental group: real acupuncture

Control group: sham acupuncture

Primary parameter: VAS for bothersomeness at 2 months after beginning of treatment

Expected mean difference and common standard deviation between two groups: 1.5 on a 0-10 VAS for bothersomeness, SD = 2.73

Drop-out rate: 20%

Sample size determination:(1)

where,

*N *: total sample size

*n*_*t *_: sample size for experimental group

*n*_*c *_: sample size for control group

r: number of experimental group

s: number of control group

λ: ratio of experimental and control sample size (λ = *n*_*t*_/*n*_*c*_)

In the trial, r = 1, s = 1 and λ = 1 were assumed, and then *N *= *n*_*t *_+ *n*_*c *_

To determine optimal sample size, we considered the following hypotheses of interest and formulas of sample size

Under hypotheses *H*_0_: μ_c _= μ_t _*H*_1_: μ_c _≠ μ_t _

sample size:(2)

μ_t _: mean of experimental group

μ_c _: mean of control group

σ^2 ^: common variance between two groups

α: probability of type 1 error (significance level)

ß: probability of type 2 error

Z_α/2_: α/2th quantile of standard normal distribution

Z_ß _: ßth quantile of standard normal distribution(3)

μ_c_-μ_t _= 1.5, σ = 2.73(Reference: Cherkin et al)[25]

Using formulas (1) above:(4)

For the equal allocation for the two groups, total sample size considering drop-out rate of 20% was calculated as 130 subjects.

### Statistical methods and analysis

All statistical analysis was performed in the principle of ITT analysis (Intention-To-Treat analysis) and PP analysis (Per-Protocol analysis). In the case of ITT analysis, we applied the LOCF (Last Observation Carried Forward Analysis) rule. For all statistical analysis, SPSS Win. Ver.14.0 and a significance level of 0.05 were used.

#### Description of baseline characteristic and homogeneity test of two groups

For the description of baseline characteristics, both demographical and clinical, for all subjects in the clinical trial, the mean with standard deviation, range with minimum and maximum for quantitative data and frequency with percentage for qualitative data are described.

For the homogeneity test of the baseline characteristics, both demographical and clinical, between the two groups, two-sample t-test for quantitative data and Chi-square test for qualitative data were performed. If there were baseline characteristics showing statistical significance and possibility of covariance, ANCOVA (analysis of covariance) was used for analysis and adjustment of baseline characteristics.

#### Efficacy

##### • Primary variable

The two-sample T-test was used for VAS for bothersomeness of low back pain at baseline and at 8 weeks for the comparison of the two groups and to determine differences from baseline. ANCOVA and two-sample T-test was used between the differences at baseline VAS and 8 week VAS for each groups. Also, a 95% confidence interval was added for all analyses.

Repeated measure two factor analysis will be used to analyze the difference and mean change among baseline, and 4, 6, 8, 12 and 24-week VAS, difference and mean change between groups, and interaction between groups and observed time. If necessary, a mixed model approach was also used.

##### • Secondary variable and others

For the variable, VAS for pain intensity, Oswestry Disability Index (ODI), SF-36, EQ-5D, repeated measure two factor analysis was used to analyze the difference and mean change among baseline, 4, 6, 8, 12 and 24-week VAS, difference and mean change between groups, and interaction between groups and observed time. If necessary, a mixed model approach was also used.

For medication use, the Chi-square test was used to analyze the different types of drugs between the groups, dose of drug and frequency of dose in a day among baseline, 4, 6, 8, 12 and 24-week VAS. Repeated measure two factor analysis was used to analyze the difference and mean change between groups, interaction between groups and observed time. If necessary, a mixed model approach was also used.

#### Safety

All adverse effects were recorded and described as frequency and percentage. For the comparison of adverse effects between groups, the Chi-square test or Fisher's exact test were performed.

### Data handling

Investigators entered the information required by the protocol into the Case Report Forms (CRFs). Non-obvious errors or omissions were entered into data query forms, which were returned to the investigational site for resolution. The data from all centers were gathered and summarized with respect to demographic baseline characteristics, effectiveness and safety observations.

## Discussion

We adopted the visual analogue scale for bothersomeness (not pain) as the primary outcome measurement. This is because the target disease of this study is chronic low back pain. According to the clinical experience, we observed that some patients with chronic pain tend to be very bothered by even a small amount of pain and others are not bothered by even moderate pain. In clinical practice, we frequently find it is more essential and effective to try to improve bothersomeness due to chronic pain than to try to reduce that pain itself.

Various randomized clinical trials that have investigated the efficacy of acupuncture tend to use different types of control groups, such as sham acupuncture, placebo acupuncture and a waiting list [8,9,26,27]. In this study, we chose placebo acupuncture (non acupuncture points) combined with sham acupuncture device. We believe that it is more valid to combine placebo acupuncture with sham acupuncture as the control group, in order to isolate the specific effects of the technique from the non-specific analgesic ones.

The results of this trial will be available in July 2010

## Competing interests

The authors declare that they have no competing interests.

## Authors' contributions

JHL were responsible for developing treatment protocol and preparation of the manuscript. HJP, HL, and IHS were responsible for the study design, provided the treatment protocols, and supervising of the protocol. MYS was contributed to the design of the study, provided the treatment protocols, and responsible for the acquisition of funding for the study. All authors have read, revised and approved the final manuscript

## Pre-publication history

The pre-publication history for this paper can be accessed here:

http://www.biomedcentral.com/1471-2474/11/118/prepub

## References

[B1] van Tulder MW Waddell G Evidence-based medicine for non-specific low back pain Best Pract Res Clin Rheumatol 2005 19 vii vix 10.1016/j.berh.2005.03.009

[B2] Frymoyer JW Back pain and sciatica N Engl J Med 1988 318 5 291 300 2961994 10.1056/NEJM198802043180506

[B3] Speed C Low back pain BMJ 2004 328 7448 1119 1121 10.1136/bmj.328.7448.1119 15130982 PMC406328

[B4] Deyo RA Weinstein JN Low back pain N Engl J Med 2001 344 5 363 370 10.1056/NEJM200102013440508 11172169

[B5] Consumer Reports How is your doctor treating you? Consumer Reports 1995 81 88

[B6] Cherkin DC Deyo RA Sherman KJ Hart LG Street JH Hrbek A Davis RB Cramer E Milliman B Booker J Mootz R Barassi J Kahn JR Kaptchuk TJ Eisenberg DM Characteristics of visits to licensed acupuncturists, chiropractors, massage therapists, and naturopathic physicians J Am Board Fam Pract 2002 15 6 463 472 12463292

[B7] National health insurance corporation Health insurance review & assessment service 2006 National health insurance statistical yearbook; Seoul 2007

[B8] van Tulder MW Cherkin DC Berman B Lao L Koes BW The effectiveness of acupuncture in the management of acute and chronic low back pain A systematic review within the framework of the Cochrane Collaboration Back Review Group. Spine (Phila Pa 1976) 1999 24 11 1113 1123 10.1097/00007632-199906010-00011 10361661

[B9] Ernst E White AR Acupuncture for back pain: a meta-analysis of randomized Pcontrolled trials Arch Intern Med 1998 158 20 2235 2241 10.1001/archinte.158.20.2235 9818803

[B10] Smith LA Oldman AD McQuay HJ Moore RA Teasing apart quality and validity in systematic reviews: an example from acupuncture trials in chronic neck and back pain Pain 2000 86 1-2 119 132 10.1016/S0304-3959(00)00234-7 10779669

[B11] Paterson C Dieppe P Characteristic and incidental (placebo) effects in complex interventions such as acupuncture BMJ 2005 330 7501 1202 1205 10.1136/bmj.330.7501.1202 15905259 PMC558023

[B12] Park J White A Stevinson C Ernst E James M Validating a new non-penetrating sham acupuncture device: two randomised controlled trials Acupunct Med 2002 20 4 168 174 10.1136/aim.20.4.168 12512790

[B13] Patrick DL Deyo RA Atlas SJ Singer DE Chapin A Keller RB Assessing health-related quality of life in patients with sciatica Spine (Phila Pa 1976) 1995 20 17 1899 1908 8560339 10.1097/00007632-199509000-00011

[B14] Revill SI Robinson JO Rosen M Hogg MI The reliability of a linear analogue for evaluating pain Anaesthesia 1976 31 9 1191 1198 10.1111/j.1365-2044.1976.tb11971.x 1015603

[B15] Carlsson AM Assessment of chronic pain. I. Aspects of the reliability and validity of the visual analogue scale Pain 1983 16 1 87 101 10.1016/0304-3959(83)90088-X 6602967

[B16] Roland M Morris R A study of the natural history of back pain. Part I: development of a reliable and sensitive measure of disability in low-back pain Spine (Phila Pa 1976) 1983 8 2 141 144 6222486 10.1097/00007632-198303000-00004

[B17] Jeon CH Kim DJ Kim DJ Lee HM Park HJ Cross-cultural adaptation of the Korean version of the Oswestry Disability Index(ODI) Journal of Korean Spine Surg 2005 12 2 145 152

[B18] Ware JE Jr Sherbourne CD The MOS 36-item short-form health survey (SF-36). I. Conceptual framework and item selection Med Care 1992 30 6 473 483 10.1097/00005650-199206000-00002 1593914

[B19] Nam BH Lee SW Testing the validity of the Korean SF-36 health survey J of the Korean Society of Health Statistics 2003 28 2 3 24

[B20] Rabin R de CF EQ-5D: a measure of health status from the EuroQol Group Ann Med 2001 33 5 337 343 10.3109/07853890109002087 11491192

[B21] Kang EJ Shin SH Park HJ Jo MW Kim NY A validation of health status using EQ-5D The Korean Journal of Health Economics and Policy 2006 12 2 19 43

[B22] Lee YH Song JY A study of the reliability and the validity of the BDI, SDS, and MMPI-D scales Korean Journal of Clinical Psychology 1991 10 1 98 113

[B23] Beck AT Ward CH Mendelson M Mock J Erbaugh J An inventory for measuring depression Arch Gen Psychiatry 1961 4 561 571 13688369 10.1001/archpsyc.1961.01710120031004

[B24] Vincent C Lewith G Placebo controls for acupuncture studies J R Soc Med 1995 88 4 199 202 7745565 PMC1295163

[B25] Cherkin DC Sherman KJ Hogeboom CJ Erro JH Barlow WE Deyo RA Avins AL Efficacy of acupuncture for chronic low back pain: protocol for a randomized controlled trial Trials 2008 9 10 10.1186/1745-6215-9-10 18307808 PMC2276474

[B26] Brinkhaus B Witt CM Jena S Linde K Streng A Wagenpfeil S Irnich D Walther HU Melchart D Willich SN Acupuncture in patients with chronic low back pain: a randomized controlled trial Arch Intern Med 2006 166 4 450 457 10.1001/.450 16505266

[B27] Witt CM Jena S Selim D Brinkhaus B Reinhold T Wruck K Liecker B Linde K Wegscheider K Willich SN Pragmatic randomized trial evaluating the clinical and economic effectiveness of acupuncture for chronic low back pain Am J Epidemiol 2006 164 5 487 496 10.1093/aje/kwj224 16798792

